# Noninvasive Optical Imaging and *In Vivo* Cell Tracking of Indocyanine Green Labeled Human Stem Cells Transplanted at Superficial or In-Depth Tissue of SCID Mice

**DOI:** 10.1155/2015/606415

**Published:** 2015-07-09

**Authors:** Vikram Sabapathy, Jyothsna Mentam, Paul Mazhuvanchary Jacob, Sanjay Kumar

**Affiliations:** ^1^Center for Stem Cell Research, Christian Medical College, Bagayam, Vellore, Tamil Nadu 632002, India; ^2^Department of Endocrine Surgery, Christian Medical College, Vellore, Tamil Nadu 632002, India

## Abstract

Stem cell based therapies hold great promise for the treatment of human diseases; however results from several recent clinical studies have not shown a level of efficacy required for their use as a first-line therapy, because more often in these studies fate of the transplanted cells is unknown. Thus monitoring the real-time fate of* in vivo* transplanted cells is essential to validate the full potential of stem cells based therapy. Recent studies have shown how real-time* in vivo* molecular imaging has helped in identifying hurdles towards clinical translation and designing potential strategies that may contribute to successful transplantation of stem cells and improved outcomes. At present, there are no cost effective and efficient labeling techniques for tracking the cells under* in vivo* conditions. Indocyanine green (ICG) is a safer, economical, and superior labelling technique for* in vivo* optical imaging. ICG is a FDA-approved agent and decades of usage have clearly established the effectiveness of ICG for human clinical applications. In this study, we have optimized the ICG labelling conditions that is optimal for noninvasive optical imaging and demonstrated that ICG labelled cells can be successfully used for* in vivo* cell tracking applications in SCID mice injury models.

## 1. Introduction

Live cell* in vivo* cell tracking can be performed by labelling cells with molecular probes that enter the cell by active/passive transport and are trapped intracellularly (e.g., direct labelling). Alternatively, cells can be labelled by overexpression of specific reporter genes that integrate into the cellular genome via viral or nonviral vectors (e.g., reporter gene labelling). Although reporter gene imaging requires genomic manipulation and poses potential safety issues, it is the preferred labelling strategy because signal generation is dependent on cell viability. Signal generated from cells labelled by either technique can then be visualized using imaging systems such as fluorescence imaging (FLI) or bioluminescence imaging (BLI). The advantages and disadvantages of each imaging system are summarized in recent study by Nguyen et al. [1]. Overall goal of molecular imaging in regenerative medicine is to enhance therapeutic efficacy and decrease cytotoxicity. Results from preclinical and clinical studies thus far suggest that cell imaging can and should be incorporated into more studies of cell transplantation in animals and humans. Cell transplantation is a very rapidly evolving technique in the field of regenerative medical applications. However, inability to track the cells* in vivo* safely and efficiently has become a major roadblock for translational applications using cell therapy. At present, a variety of techniques used for* in vivo* imaging include magnetic resonance imaging [2], reporter gene labeling via fluorescence [3] and bioluminescence imaging [4], single-photon emission computed tomography (SPECT) [5], positron emission tomography (PET) [6], ultrasound [7], nanoparticles [8], quantum dots [9], and fluorescent dyes [10]. In 2004, Frangioni and Hajjar first presented the 8 ideal characteristics of imaging technology for stem cell tracking under* in vivo* condition [11]. Over the years, until now, no proper imaging technology has been developed that can be rendered suitable for translational applications. In 2010, Boddington et al. clearly described the efficient tracking of (indocyanine green) ICG labeled cells by means of noninvasive optical imaging technique under* in vitro* conditions [12]. In 1955 Kodak Research Laboratory first developed ICG for near infrared photography. In 1959 FDA approved the ICG for human diagnostic applications [13]. ICG has been employed in clinical applications such as determination of cardiac output, liver function diagnostics, ophthalmic angiography, sentinel lymph node detection in oncology, neurosurgery, coronary surgery, vascular surgery, lymphography, liver surgery, laparoscopy, reconstructive microsurgery, phototherapy, and dyeing [14–17]. ICG is a tricarbocyanine dye, exhibiting peak absorbance and emission at 780 nm and 830 nm, respectively [18]. The absorption and fluorescence spectra of ICG are in the near infrared region. Both depend largely on the solvent used and the concentration. ICG absorbs mainly between 600 nm and 900 nm and emits fluorescence between 750 nm and 950 nm [13]. The large overlapping of the absorption and fluorescence spectra leads to a marked reabsorption of the fluorescence by ICG itself. The fluorescence spectrum is very wide. Its maximum values are approximately 810 nm in water and approximately 830 nm in blood [14]. For medical applications based on absorption, the maximum absorption at approximately 800 nm (in blood plasma at low concentrations) is important [13]. In combination with fluorescence detection, lasers with a wavelength of around 780 nm are used. At this wavelength, it is still possible to detect the fluorescence of ICG by filtering out scattered light from the excitation beam [14]. ICG has somewhat bizarre light absorption behavior as a function of concentration because it tends to aggregate in water at high concentrations. This means that the effective absorption does not increase linearly with increasing concentration. Furthermore, ICG tends to degrade with exposure to light. The photodegradation is mitigated when ICG is bound to albumin, but it still proceeds slowly (days). The photodegradation is also concentration dependent. ICG is metabolized microsomally in the liver and only excreted via the liver and bile ducts; since it is not absorbed by the intestinal mucous membrane, the toxicity can be classified as low [15]. Administration is not without risks during pregnancy. It has been known in the literature that ICG decomposes into toxic waste materials under the influence of UV light, creating a number of still unknown substances [15]. Past study, however, shows that ICG (the substance without UV effect) is basically, as such, of only minor toxicity [15]. The intravenous LD_50_ values measured in animals are 60 mg/kg in mice and 87 mg/kg in rats. Occasionally, in one out of 42,000 cases, slight side-effects occur in humans such as sore throats and hot flushes. Effects such as anaphylactic shock, hypotension, tachycardia, dyspnea, and urticaria only occurred in individual cases; the risk of severe side-effects rises in patients with chronic kidney impairment [17]. The frequencies of mild, moderate, and severe side-effects were only 0.15%, 0.2%, and 0.05%. For the competitor substance fluorescein, the proportion of people with side-effects is 4.8%. This study was designed to investigate the potential of ICG labeled cells for* in vivo* tracking applications. In this study we have used human placental mesenchymal stromal cells (hPDMSCs), human Wharton's Jelly MSCs (WJMSCs), and human induced pluripotent stem cells (iPSCs) derived neurospheres (iNSCs) for labeling with ICG and tracking the transplanted cells using skin injury and spinal cord injury SCID mice models. The tracking of the labeled transplanted cells was carried out using IVIS preclinical* in vivo* imaging system. IVIS system has preset near infrared (NIR) excitation (710–760 nm) and emission (810–875 nm) pass band filters to evaluate the ICG labeling. Continued application of molecular imaging for regenerative cell therapies will be critical for its successful applications.

## 2. Materials and Methods

### 2.1. Preparation of ICG Stock Solution

The ICG solution used in this study was prepared by dissolving ICG powder (laser grade I-25; Sigma-Aldrich Co., St. Louis, MO) in alpha modifications of minimum essential medium (alpha MEM, Lonza) with resulting stock solutions at concentrations of 0.5, 1, 2, and 2.5 mg/mL.

#### 2.1.1. Optical Imaging

*In vivo* and* in vitro* fluorescence imaging was performed with an IVIS spectrum CT imaging system (Xenogen, Perkin Elmer, MA, USA). The optical imager is an integrated fluorescence system (400–900 nm) that is composed of a light-tight specimen chamber (dark box) and a charge-coupled device (CCD) camera. To minimize electronic background and maximize sensitivity, the CCD camera is thermoelectrically cooled to −90°C. All* in vitro* experimental samples were imaged in 1 mL of cell culture media in a 24-well plate. All images were acquired using the filter setting preset for ICG with a background wavelength at 665–695 nm, an excitation wavelength at 710–760 nm, and an emission wavelength set at 810–875 nm. Consistent illumination parameters were used for all NIR fluorescent acquisitions. For all experiments, the field of view (FOV) was adjusted to focus on entire area, exposure time was adjusted to avoid saturation of signal, f/stop was adjusted automatically by software, lamp voltage was set to “high,” and binning was kept on medium. Following acquisition, all images were normalized to units of average efficiency, displayed in the same scale of fluorescent intensity, and analyzed using the Living Image 4 software (Xenogen, Perkin Elmer, MA, USA).

### 2.2. ICG Labeling of Human Stem Cells

In order to estimate the optimal concentration for labeling the cells, 5 × 10^4^ hPDMSCs were seeded per well of 6-well plate. When the seeded cells reached the 70–80% confluency, the cells were subjected to trypsinization and incubated with different ICG concentrations (0.1 mg/mL–2 mg/mL) in alpha MEM culture media. For ICG labeling, the cells were incubated for 30 minutes at 37°C. After labeling, the excess dye was removed by washing the cells twice with DPBS and the cells were further cultured in complete *α*MEM (alpha minimum essential media) media. The cells that were not labeled with ICG were used as a control. In order to measure the photon emission from the labeled cells, IVIS spectrum CT imaging system (Xenogen, Perkin Elmer, MA, USA) was employed.

#### 2.2.1. Optimization of the Cell Labeling Protocol

Human stem cells were labeled with different ICG concentrations range of 0.1 mg/mL–2 mg/mL and incubated for 30 minutes at 37°C. Cell viability by trypan blue dye exclusion test and apoptosis assays was performed to establish the viability of the labelled cells.

#### 2.2.2. *In Vivo* Longitudinal Follow-Up Studies

Human stem cells were labeled with the optical labeling protocol. Labeling efficiency was determined based on experiments from the above protocol. 1 × 10^5^ cells were injected in superficial or injured spinal cord injury site and each mouse was imaged every day for 21 days until the fluorescence signal matched that of the nonlabeled control cells. Cohorts of 6 mice were in each group and all* in vitro* optical imaging experiments were performed in triplicate.

#### 2.2.3. Optical Imaging Data Analysis

For quantitative analyses of optical imaging data, regions of interest (ROI) were placed on each ICG test sample (control side versus ICG labeled cells injected site). Quantitative measures of fluorescence intensity of labeled cells in the test mice site were normalized to units of average efficiency, displayed in the same scale of fluorescent intensity, and analyzed using the Living Image 4 software (Xenogen, Perkin Elmer, MA, USA). The unit of “efficiency” represents the fractional ratio of emitted photons per incident excitation photon (Xenogen, Perkin Elmer, MA, USA).

### 2.3. Collection of Human Placenta Samples

Human placenta tissue (biological waste material following delivery) was collected after obtaining written consent from the patients undergoing full-term pregnancy elective caesarean. The study was carried out after Institutional Review Board (IRB) approval from Christian Medical College, Vellore, India.

### 2.4. Animals

Black SCID mice (B6.CB17-prkdcScid/SzJ) were used in this study. The mice were purchased from Jackson Laboratory (Bar Harbor, ME, USA). Institutional animal ethics committee approved the experiments. The study was carried out in accordance with the institutional guidelines for animal care of Christian Medical College, Vellore, India.

### 2.5. WJMSCs

Human WJMSCs were derived as previously described earlier by Sabapathy et al. [19, 20].

### 2.6. Placental MSCs

Isolation of human placental MSCs was carried out as previously described [21].

### 2.7. iNSCs (Neurospheres Derived from hiPSCs)

The virus-free integration-free safer iPSCs were generated using nucleofection protocol (Lonza). We have optimized the protocol for efficient generation of human iPSCs from placental MSCs on autologous feeders. For neural induction, iPSCs derived embryoid bodies were treated with neural induction media (Stem Cell Technologies, BC, Canada) supplemented with rock inhibitor (Y-27632). Further, these were plated onto PLO/laminin coated plates for further differentiation.

### 2.8. Cytotoxicity Analysis

The preliminary cytotoxicity analysis of the ICG treated cells was carried out using trypan blue dye exclusion test [22]. Cell viability testing by trypan blue tests was performed on all cells before and after the labeling procedure to verify viability. Cells were counted in hemocytometer and used for subsequent optical imaging studies.

### 2.9. Apoptosis Analysis

Apoptosis analysis was carried out as previously described earlier by Sabapathy et al. [19, 21].

### 2.10. Skin Injury Model

For skin injury model, an area of 1 cm × 1 cm dorsal skin was removed in order to create a wound. About 1 × 10^6^ ICG (0.2 mg/mL) labeled WJMSCs seeded onto decellularized amniotic membrane scaffold were sutured over the site of injury [19].

### 2.11. Spinal Cord Injury Model

The spinal cord injury mice model was developed by creating contusion spine injury after laminectomy between T9 and T12. The lower thoracic spine injured mice were transplanted with 1 × 10^6^ ICG labeled cells (hPDMSCs/iNSCs). For labeling, the cells after trypsinization were treated with 0.2 mg/mL ICG and incubated at 37°C for 30 min. The cells were washed with DPBS twice and resuspended with 25 *μ*L of PBS before transplantation.

### 2.12. *In Vivo* Noninvasive Imaging

The total flux emitted from the ICG labeled cells after transplantation was monitored using IVIS spectrum CT imaging system (Xenogen, Perkin Elmer, MA, USA).

### 2.13. Statistical Analysis

Sigma Plot V11.0 software was used for statistical analysis. The data are expressed as mean ± standard deviation (SD). Statistical analysis between multiple groups was estimated using analysis of variance (ANOVA) test followed by Tukey's method. The values with *p* < 0.05 were interpreted to be significant.

## 3. Results

### 3.1. ICG Labeling of Cells

**Figure 1:**
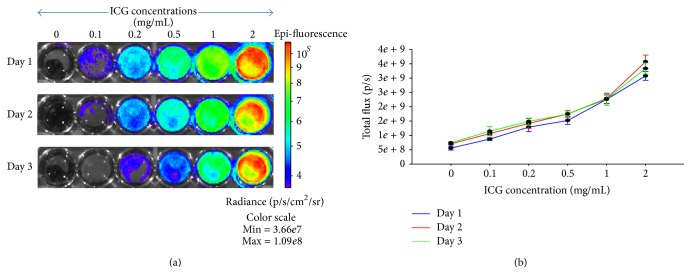
ICG concentration optimization: (a) heat map representing the total flux emitted by placental MSCs labeled with different concentrations of ICG; (b) graphical representation of total flux emitted at different concentrations with respect to duration of culture.

Initially, we had focused on standardizing the ICG concentration for effective cell labeling (Figure 1) with minimal cytotoxicity tested by cell survival and cellular apoptosis assays. The human placental MSCs were seeded in the 24-well plates; when confluency reached about 70–80% cells were trypsinized and labeled by treating with different concentrations of ICG (0.1 mg/mL–2 mg/mL). Our data suggests that the total flux emitted by the labeled cells was directly proportional to the concentration of ICG. There was no significant decrease in the total flux of the labeled cells over the period of 3 days. Since* in vitro* cultured fetal MSCs are rapidly dividing cells, the observation could not be carried out for more than 3 days as a result of overconfluency of the cells. Further, we noted that after trypsinization in 1 : 2 ratio the ICG fluorescence levels were getting diluted and significantly lower fluorescence was recorded.

### 3.2. Cytotoxicity by Trypan Blue Dye Exclusion Experiment

**Figure 2:**
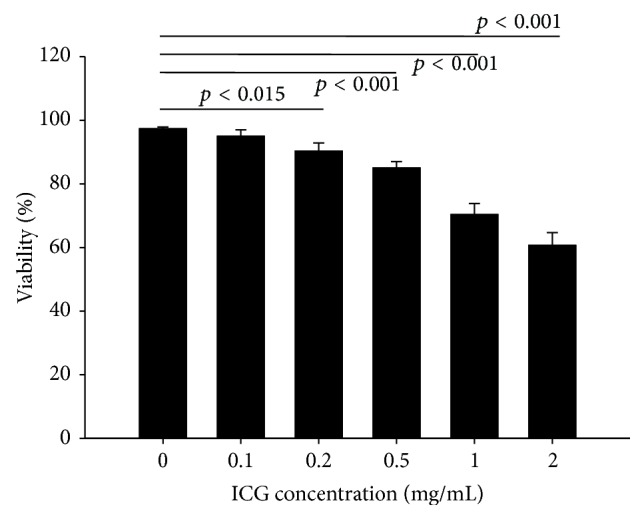
Cellular cytotoxicity: graphical representation of cytotoxicity assay of placental MSCs labeled cells at various concentrations.

In order to access the cytotoxicity of the cells, simple trypan blue dye exclusion test was carried out (Figure 2).

Human placental MSCs were subjected to varying concentrations of ICG (0.1–2 mg/mL) and the viability of the cells was monitored after 24 hrs. Our viability data suggest that the viability of the cells was inversely proportional to the ICG concentrations (Figure 2). The results obtained from our data were in accordance with previous studies representing that ICG concentration of over 0.25 mg/mL significantly decreased the viability of the cells [23]. We choose 0.2 mg/mL concentration of ICG for safer labeling of cells without compromising on the* in vivo* signal quality for the purpose of transplantation.

### 3.3. Apoptosis Analysis by FACS

For quantification of apoptotic cells, originated from cytotoxic effects of 0.2 mg/mL and 0.5 mg/mL ICG concentration on incubating hPDMSCs, cells were stained with 7AAD and PE labelled annexin V kit (BD Pharmingen, CA, USA) before acquiring and analysing the percentage of apoptotic cells through BD FACS CALIBUR flow cytometry.

**Figure 3:**
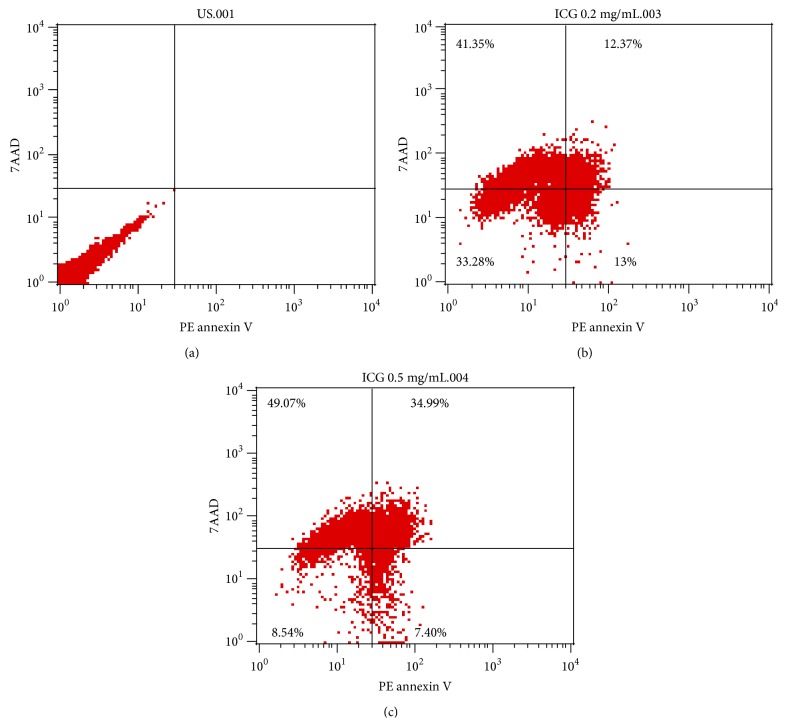
Apoptosis analysis of hPDMSCs: (a) unstained; (b) 0.2 mg/mL ICG; (c) 0.5 mg/mL.

Additionally, apoptosis analysis was carried out for stringent cytotoxicity effect evaluation. The data suggested that placental MSCs treated with ICG concentrations of 0.5 mg/mL (Figure 3(c)) exhibited significantly greater number of cells undergoing apoptosis compared to cells treated with 0.2 mg/mL ICG concentrations (Figure 3(b)). Implying higher ICG concentrations can leave detrimental effects on the viability and/or general health of the cells.

#### 3.3.1. Longitudinal Optical Imaging of ICG Labelled hPDMSCs, WJMSCs, and iNSCs Derived from hiPSCs

Far-red fluorescence labelling of hPDMSCs with ICG at a concentration of 0.2 mg/mL after a 30 min incubation time at 37°C revealed that the fluorescence signal slowly decreased over time when assayed* in vitro* on days 1, 2, 3, and so on compatible with slow release of the contrast agent from the cells. The signal decrease was quantified from the emitted photons in the region of interest (ROI) after labelling and was equivalent to controls at 120 h after labelling in the 0.2 mg/mL ICG concentration well (Figure 1). The fluorescence signal at 1 h, 24 h, 48 h, and 72 h was significantly higher compared to precontrast data (*p* < 0.05). When assayed* in vitro*, the signal at 120 h after labelling was not significantly different from baseline (*p* > 0.05) in the 0.2 mg/mL ICG concentration well.

Longitudinal studies of human PDMSCs and WJMSCs labelled with 0.2 mg/mL of ICG for 30 min at 37°C revealed similar fluorescence signal kinetics compared to labelled hiPSCs. The cells fluorescence signal was significantly elevated at 1, 24, and 48 h (*p* < 0.05) compared to the controls.

### 3.4. *In Vivo* Imaging of ICG Labeled Cells

#### 3.4.1. Skin Injury

**Figure 4:**
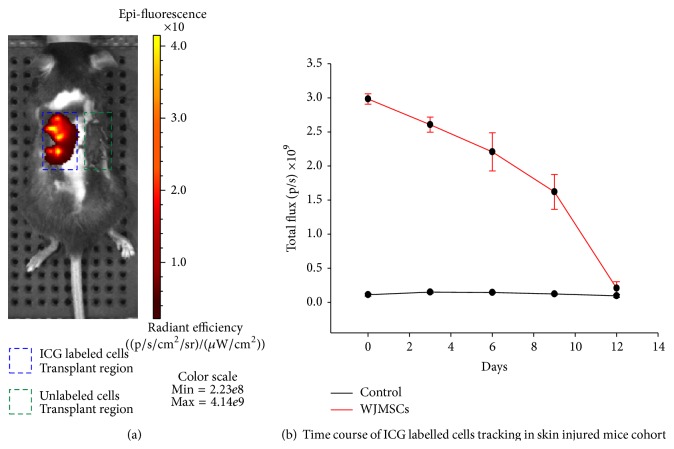
(a) Skin injury, ICG labelled WJMSCs (ICG +/−), seeded onto decellularized amniotic membrane scaffold grafted over the site of injury. (b) ICG far-red fluorescence signal quantification from noninvasive time course imaging of ICG labelled cells that were tracked by noninvasive optical imaging on day 1, day 3, day 6, day 9, and day 12 following skin injury in SCID mice.

For skin injury model, the cells were labeled after trypsinization and incubated at 37°C for 30 minutes. The ICG labeled cells (1 × 10^6^) were seeded onto decellularized amniotic membrane. After 24 hrs, amniotic membrane containing the labeled WJMSCs was grafted over the skin injured mice model. The total flux from the labeled cells was monitored daily. The flux emitted from the superficial tissue injury model deteriorated gradually and lasted for about 2 weeks (Figure 4). We have also tested the direct injection of ICG labeled cells (1 × 10^6^) at the site of injury and the duration of photon emission remained the same.

### 3.5. Spinal Cord Injury in SCID Mice

#### 3.5.1. Fluorescence

**Figure 5:**
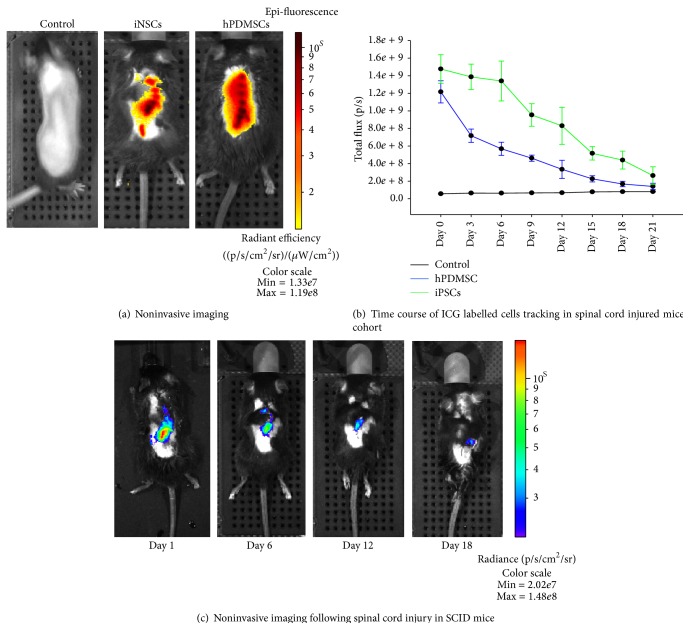
(a) Spinal cord injury, ICG labeled iNSCs/hPDMSCs transplanted at the site of injury. (b) Far-red fluorescence data quantification of ICG labelled hPDMSs and iNSCs derived from iPSCs cells that were* in vivo* tracked for 21 days following transplantation in spinal cord injury of SCID mice. (c) Representative images on days 1, 6, 12, and 18 of* in vivo* ICG labelled hPDMSCs cells tracking following spinal cord injury in SCID mice.

The fluorescence signal from ICG labelled hiPSCs was significantly higher compared to the fluorescence signal of hPDMSCs at day 1 (*p* < 0.05), day 3 (*p* < 0.05), day 6 (*p* < 0.05), and day 9 (*p* < 0.05) (Figure 5(b)).

#### 3.5.2. Viability Testing

Trypan blue exclusion testing showed no significant difference in viability between labelled iNSCs derived from hiPSCs or labeled hPDMSCs compared to unlabeled controls. All groups exhibited viabilities greater than 95%, which were not significantly different between experimental groups (*p* > 0.05) (Figures 2 and 5).

In the case of deep tissue wound model, the contusion injury was created at the lower thoracic spinal cord region of the mice. Just before transplantation, the cells were labeled with ICG (0.2 mg/mL) and resuspended in about 25 *μ*L of PBS. The photon emission from the transplanted cells (iNSCs/hPDMSCs) was measured every alternate day. Our data indicate that signals from the deep tissue model were absolute and lasted for about three weeks (Figure 5).

## 4. Discussions

The efficacy of any cell therapy depends on the interaction of many different factors such as disease etiology, cell type, delivery route, cell retention/engraftment, activation of resident cells, or functional integration. In order to optimize cell therapies, we need to improve our understanding of how these factors interact* in vivo*. Recent stem cells imaging studies have highlighted some of the barriers to clinical translation in using either adult or pluripotent stem cells: (1) limited cell engraftment, cell survival, and cellular proliferation; (2) poor cell differentiation and cell maturation; (3) immunogenicity with allogeneic cell transplantation; and (4) potential tumorigenicity with pluripotent stem cell derivatives.

Present study is innovative with regard to optimization of conditions for safe ICG labelling of cells with minimal toxicity for cell proliferation, cellular apoptosis, and* in vivo* tracking of transplanted human stem cells in SCID mice at least by twofold. (1) To the best of our knowledge, this is the first* in vivo* stem cell tracking study that utilized the FDA-approved fluorochrome ICG for the labelling of human stem cells after trypsinization of cells (most of the previous studies have used the labelling of cells in the 2D culture dishes and then trypsinized the cells; this actually reduces the ICG signal dramatically in our experiment) such as hPDMSCs, WJMSCs, and iNSCs derived from hiPSCs. (2) These* in vitro* labelled cells were transplanted in superficial or deep tissue injury sites in SCID mice and tracked* in vivo* by noninvasive optical imaging. After generation of virus-free, integration-free iPSCs, we wanted a safer technique to monitor the cells* in vivo*. Previous data from Boddington et al. is the first comprehensive report indicating the potential of ICG labeled cells for noninvasive cell tracking applications using optical imaging. In this study we have further optimized the cell labeling protocol and clearly demonstrated that ICG labeled cells can be efficiently used for tracking the cells even from deep tissue regions. ICG labeling of cells and tracking of cell using optical interface successfully meet all the criteria laid down by Frangioni and Hajjar for ideal stem cell tracking technology during clinical trials [11]. Moreover, the preexisting FDA approval of ICG for human clinical applications further renders the use of ICG more attractive for translational research with less safety concern compared to other fluorescent dyes available in the market. The labeling technique using ICG is very easy and economical, although the initial cost of optical imaging interface is high. The operational and additional overhead maintenance cost is very minimal. Furthermore image acquisitions and analysis of the* in vivo* data are very rapid and cost efficient. Further, noninvasive efficient cell tracking feature of this application makes this application much more appealing than the existing methods which is cumbersome and costly and requires repeated invasive procedures. Thus it is important to focus on future role of molecular imaging in defining safety and efficacy for clinical implementation of stem cell therapies.

In this study, we have only looked at 3 different cell types hPDMSCs, iNSCs, and WJMSCs. Further horizontal studies are required to evaluate the effect and labeling conditions required for a specific cell type. According to our data (Figures 4 and 5), we could track the labeled cells for only 3 weeks. Hence we need to develop the labeling efficacy and regulate the degradation kinetics of ICG using concomitant agents/technique.

It is difficult to comprehend that although ICG was developed several decades ago and has been routinely used in several clinical applications, the use of this dye for cell tracking applications has been discovered recently. Through our research we have tried to highlight the importance of ICG in translational applications.

## 5. Conclusions

Our proof of principle in the current study productively demonstrates that ICG labeling can be used to track the cells not only from the superficial tissue site but also from deep tissue site after transplantation. Based on the previous studies, we propose that ICG based labeling techniques hold great potential in* in vivo* cell tracking during clinical applications. However, further studies are required to optimize and validate the findings in different animal as well as human models.
